# Comparison of Soil Total Nitrogen Content Prediction Models Based on Vis-NIR Spectroscopy

**DOI:** 10.3390/s20247078

**Published:** 2020-12-10

**Authors:** Yueting Wang, Minzan Li, Ronghua Ji, Minjuan Wang, Lihua Zheng

**Affiliations:** 1 Key Laboratory of Modern Precision Agriculture System Integration Research, Ministry of Education, China Agricultural University, Beijing 100083, China; wyt_study@cau.edu.cn (Y.W.); limz@cau.edu.cn (M.L.); jessic1212@cau.edu.cn (R.J.); 2 Key Laboratory of Agricultural Informatization Standardization, Ministry of Agriculture and Rural Affairs, China Agricultural University, Beijing 100083, China; minjuan@cau.edu.cn

**Keywords:** Vis-NIR spectroscopy, soil total nitrogen, machine learning, deep learning, convolutional neural network

## Abstract

Visible-near-infrared spectrum (Vis-NIR) spectroscopy technology is one of the most important methods for non-destructive and rapid detection of soil total nitrogen (STN) content. In order to find a practical way to build STN content prediction model, three conventional machine learning methods and one deep learning approach are investigated and their predictive performances are compared and analyzed by using a public dataset called LUCAS Soil (19,019 samples). The three conventional machine learning methods include ordinary least square estimation (OLSE), random forest (RF), and extreme learning machine (ELM), while for the deep learning method, three different structures of convolutional neural network (CNN) incorporated Inception module are constructed and investigated. In order to clarify effectiveness of different pre-treatments on predicting STN content, the three conventional machine learning methods are combined with four pre-processing approaches (including baseline correction, smoothing, dimensional reduction, and feature selection) are investigated, compared, and analyzed. The results indicate that the baseline-corrected and smoothed ELM model reaches practical precision (coefficient of determination (R^2^) = 0.89, root mean square error of prediction (RMSEP) = 1.60 g/kg, and residual prediction deviation (RPD) = 2.34). While among three different structured CNN models, the one with more 1 × 1 convolutions preforms better (R^2^ = 0.93; RMSEP = 0.95 g/kg; and RPD = 3.85 in optimal case). In addition, in order to evaluate the influence of data set characteristics on the model, the LUCAS data set was divided into different data subsets according to dataset size, organic carbon (OC) content and countries, and the results show that the deep learning method is more effective and practical than conventional machine learning methods and, on the premise of enough data samples, it can be used to build a robust STN content prediction model with high accuracy for the same type of soil with similar agricultural treatment.

## 1. Introduction

Soil total nitrogen (STN) has a significant impact on plant growth [1,2], and predicting STN content is vital for crop production as well as income generation for farmers. Spectroscopic techniques have the advantages of fast detection speed, low cost, and non-destructive [3], and it has been widely used in evaluation of crop yield [4,5], detection of fruit maturity [6,7,8], monitoring plant diseases and pests [9,10], and other areas of agricultural production. The spectral absorptions are associated with the stretching and bending of bonds forming the O-H, N-H, and C-H groups in the visible-near-infrared spectrum (Vis-NIRS) bands [11], so Vis-NIRS has been one of the most primary means of predicting STN content. Building predictive models is an essential link for identifying and predicting STN content by Vis-NIRS, the selection and application of models will directly affect the effectiveness of prediction [12,13].

Traditional modeling approaches to evaluate STN content often involve complicated data preprocessing since there exist a nonlinear relationship between STN and soil spectra. Among them, partial least square (PLS) and principal component regression (PCR) are the most common modelling approaches for quantitative spectroscopy analyses in soils [14,15]. Veum et al. [16] predicted total nitrogen (TN) using a large, regional dataset of in situ profile DRS spectra and compare the performance of traditional PLS analysis, PLS on external parameter orthogonalization (EPO) transformed spectra (PLS-EPO), PLS-EPO with the Bayesian Lasso (PLS-EPO-BL), and covariate-assisted PLS-EPO-BL models. The results shown the PLS-EPO transformation dramatically improved model performance relative to PLS alone, reducing RMSEP by 53% for TN. Support vector regression (SVR) is another common conventional machine learning modeling method in this case. Debanen et al. [17] selected SVR to predict soil properties from texturally homogeneous samples (loamy sand), the results shown that Vis-NIRS is suitable for the prediction of properties of texturally homogeneous samples. The best results were obtained for N content prediction using the full dataset with cross-validation (R^2^ = 0.81, root mean square error of cross-validation (RMSECV) = 0.01, RPD = 2.20) and with an independent validation dataset (R^2^ = 0.73, RMSEP = 0.03, RPD = 1.22).

In addition, with the development of nonlinear modeling methods and machine learning technology, random forest (RF) and neural network have achieved good results in STN content prediction. RF method shows great potential to deal with the nonlinear relationship between soil total nitrogen and spectrum. By establishing a RF model, Zhang et al. [18] determined the spatial distribution of STN and investigated the importance of different predictors. This work employed 21 predictors (including the original bands (O), normal spectral indices (S), red-edge indices (R), and environment variables (E)) to estimate the spatial distribution of the STN content. The results suggested that the performance of the prediction model could be improved by providing enough types of suitable predictors. Neural network has been becoming a popular method used in STN content prediction due to its good performance, including BP neural network [13] and extreme learning machine (ELM) [19]. Li et al. [20] used uninformative variable elimination (UVE) to extract characteristic wavelengths, and then ELM was applied to establish the calibration models with full spectra and characteristic wavelengths. They concluded that the UVE-ELM model performed better (R^2^ is 0.9408, RMSEP is 0.0075 and residual prediction deviation (RPD) is 2.97).

However, the above conventional methods are more suitable for smaller sample size, and usually a set of complicated data preprocessing and/or spectral transformation are required before modeling. Misuse of data preprocessing or spectral transformation may introduce artifacts of interferences or remove useful information patterns and result in worse model performance [21].

With the rise of deep learning which is based on the improvement and innovation of neural network technology, researchers are introducing deep learning method to predict soil properties. Convolutional neural networks (CNNs) is one of the most popular deep learning approach, and it can discover intricate structures in high-dimensional data but reducing the need from prior knowledge and human effort in feature engineering [22]. Zhang et al. [21] proposed a method called DeepSpectra which incorporated Inception module based on a CNN, and tested the performance on four open accessed visible and near infrared spectroscopic datasets (corn, tablet, wheat, and soil). The paper showed that DeepSpectra approach provided better results than conventional linear and nonlinear calibration approaches in most scenarios. Riess et al. [23] developed and implemented three one-dimensional CNN: LucasCNN, LucasResNet, and LucasCoordConv, to carry out soil texture classification. They evaluated the performance of the CNN approaches and compared them by testing the freely available LUCAS topsoil dataset. The results showed that the LucasCoordConv achieved the best performance regarding the average accuracy. We can infer from the above successful studies that deep learning modeling method can be used to establish STN content prediction model when a massive soil sample data is available.

From a practical point of view, different modeling approaches may be suitable for different application conditions and lead to different performances. In order to investigate which modeling approach can get higher accuracy without complicated spectral transformation and clarify model effectiveness based on different data characteristics, several mainstream conventional STN content modeling methods, along with deep learning modeling techniques, are implemented and compared in this paper, and their effectiveness are explored according to data feature. The objectives of this paper are: (1) establish reasonable STN prediction models based on Vis-NIRS using both conventional machine learning and deep learning methods with LUCAS topsoil dataset; (2) compare the performance of the built models to find which method suitable for predicting STN content with different conditions; and (3) evaluate the effectiveness and applicability of the models due to data characteristics. Based on the results, we believe that this paper can help the researchers and engineers to quickly build a robust and effective STN content prediction model for their own soil samples.

## 2. Materials and Methods

**Figure 1:**
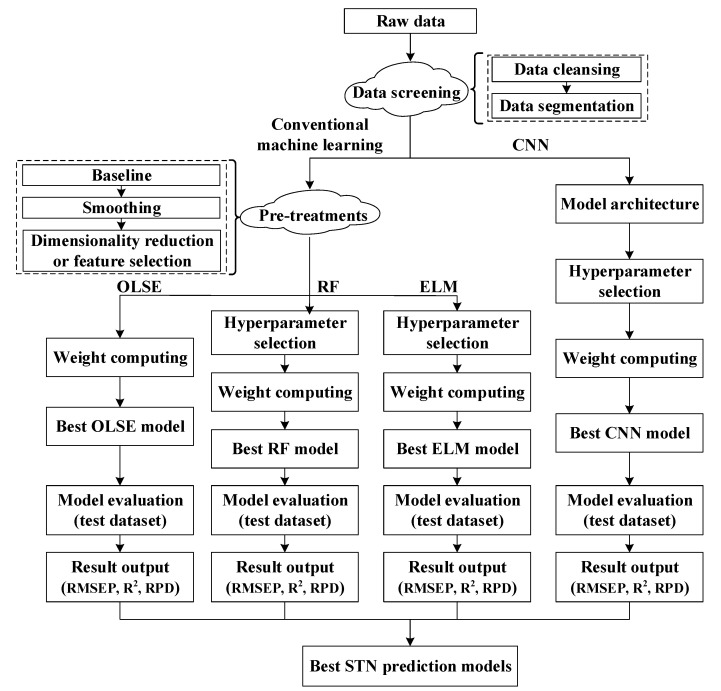
Workflow for soil total nitrogen (STN) prediction model based on visible-near-infrared spectrum (Vis-NIRS).

Figure 1 is the workflow for STN prediction models based on Vis-NIRS. Firstly, the raw data is read-in, and data screening is carried out to cleanse and segment the dataset. Secondly, the datasets are subjected to preprocessing in conventional machine learning approaches. Finally, through each modeling process, the best model can be obtained from each specific method according to performance evaluation. The following sections expand upon the workflow in greater detail.

All the models are implemented on the Python platform using Keras and Scikit-learn library. The process is run on a Linux server (version is Ubuntu 16.04) with 64 GB of RAM, and an Nvidia Geforce GTX 1080Ti graphics card with 11 GB of RAM.

### 2.1. Dataset Description

In this paper, we use the open accessed Land Use/Cover Area Frame Statistical Survey (LUCAS) Soil dataset. The project collected 19,036 samples of topsoil and determined their soil parameters and measured their Vis-NIR spectra after oven-dried. The published dataset consists of soil properties (clay, silt and sand content, coarse fragments, pH, organic carbon content, nitrogen, etc.) and Vis-NIRS absorbance. The spectra covered a range from 400 to 2499.5 nm with a resolution of 0.5 nm [23]. Table 1 shows the description of the LUCAS Soil dataset and it is available at: https://esdac.jrc.ec.europa.eu/content/lucas-2009-topsoil-data#tabs-0-description=0.

### 2.2. Data Preparation and Pre-Processing

#### 2.2.1. Data Cleansing and Data Segmentation

Given the experimental environment changes and variability of sample origins, there exist various noise signals and artifacts derived from sample pretreatment, instrument, and experiment. To reduce noise, data cleansing was carried out and those samples with STN = 0 g/kg were removed. Accordingly, 19,019 samples were obtained as raw experimental data to be used to establish the prediction models.

In most cases, just two data sets are commonly needed to calibrate and validate model in conventional machine learning methods. In this paper, in order to maximize the utilization of reasonably distributed data to obtain the most reliable model, instead of random data division, the samples were sorted in descending order according to their STN content at first. Then a set of data was then extracted at regular intervals (every 5 samples) as the test subset (3804 samples), and the other samples as train subset (15,215 samples). These two data subsets were used in conventional methods-based modeling. However, when CNN modeling was investigated, the above train subset was randomly partitioned into two subsets: the validation subset (25% of the data, 3804 samples), and the train subset (75% of the data, 11,411 samples).

#### 2.2.2. Baseline Correction and Smoothing

Preprocessing is usually required to enhance calibration model accuracy by reducing un-modeled variations such as instrumental and experimental artifacts [24,25]. Baseline correction and smoothing are the typical preprocessing approaches for Vis-NIRS analysis. In this paper, we selected asymmetric least squares baseline correction method (AsLS) and Savitzy–Golay smoothing method (SGS) to remove the noise from the raw spectral data.

Eliser et al. [26] proposed the AsLS approach, and the target function is presented in Equation (1).

(1) $$z=arg\underset{z}{\min}\left\{\underset{i}{\sum }w_{i}{\left(y_{i}-z_{i}\right)}^{2}+\lambda \underset{i}{\sum }{\left(\Delta^{2}z_{i}\right)}^{2}\right\}$$

where z represents the fitting spectrum; $y_{i}$ is the raw data at wavelength of i nm; $z_{i}$ is the fitting information at wavelength of i nm, and $w_{i}$ is the weight at wavelength of i nm. λ is an adjustment coefficient, and $\Delta^{2}z_{i}$ is differential operation and presented in Equation (2).

(2) $$\Delta^{2}z_{i}=\left(z_{i}-z_{i-1}\right)-\left(z_{i-1}-z_{i-2}\right)=z_{i}-2z_{i-1}+z_{i-2}$$

When $y_{i}$ is much higher than $z_{i}$, let $w_{i}=p$; when $y_{i}$ equal to or less than $z_{i}$, $w_{i}=1-p$. Here p is introduced as another adjustment coefficient. So, there are two free parameters to tune: λ and *p*. In general, p takes values in the range (0.001–0.1), and λ takes values in the range (102–109). p is set to 0.01 and λ is set to 10^9^ in this paper.

SGS convolution smoothing algorithm is one of the most commonly used spectral smoothing methods. The algorithm uses mathematical methods to establish polynomial regression of local interval data and evaluate its value in the center of approximate window. It not only inherits the advantages of moving average algorithm for fast and simple denoising, but also can effectively retain the relative maximum, minimum, and changeable information of the signal. Compared to traditional filters, SGS is faster and can handle missing data quite well [27]. In this work, the window size is set to 9 and uses a third order polynomial.

#### 2.2.3. Dimensionality Reduction

Conventional machine learning approaches attempt to assign weight to each feature or band (in the case of Vis-NIRS) based on how much information is contained in each feature or band. However, they do not perform well in high dimensional spectral data, so that dimensionality reduction is required. Principal components analysis (PCA) is one of the most popular and effective dimension reduction methods used to explore variation in complex datasets [28].

PCA can transform a high dimensional dataset into a lower dimensional orthogonal feature set while retaining maximum information from the original high dimension dataset. To this goal, the samples (*x_i_*) are projected with *W^T^x_i_* in new hyperplane (*W*), and the location of the projection point determine the information of the new samples. The optimization function (*Target*) is expressed as Equation (3).

(3) $$Target=\;\underset{w}{\max}\;\;tr\left(W^{T}XX^{T}W\right)$$

In the formula,

(4) $$W^{T}\;W=\;I$$

where *I* is the unit matrix, *XX^T^* is the covariance matrix. Eigen-decomposition is performed on the covariance matrix to have the eigenvectors and corresponding eigenvalues, and the eigenvalues are then sorted in descending order. The required numbers of eigenvalues are taken, and then form a new eigenvector. The new eigenvector is the solution of PCA. In this paper, the number of principal components set to 2–50.

#### 2.2.4. Feature Selection

Feature selection is another way to reduce a high-dimensional feature space, and it ranks the features by a metric and eliminates all features that do not achieve an adequate score. Usually, feature selection methods are classified into three general groups: filter, wrapper, and embedded methods [29]. Because wrapper and embedded methods are related to prediction models, we instead chose the mutual information (MI) method to select feature, which falls under the category of the filter.

With the MI method, the feature subset can be obtained by calculating the mutual information between the features themselves and between the features and the class variables [30]. To measure the value of mutual information, information entropy is incorporated. Given a discrete random variable *X*, the entropy of *X* is denoted as H(X) (Equation (5))

(5) $$H\left(X\right)=-\underset{x\in X}{\sum }p\left(x\right)\log_{2}p\left(x\right)$$

where p(x) is the prior probability of *X*. MI has different expressions, and this paper chooses the expression (Equation (6)) proposed by Ross [31].

(6) $$MI\left(X,Y\right)=\Psi \left(k\right)-\langle \Psi \left(n_{x}+1\right)+\Psi \left(n_{y}+1\right)\rangle +\Psi \left(N\right)$$

where *Ψ(.)* is the digamma function, $\langle X\rangle =N^{-1}\sum_{i=1}^{N}x_{i}$, the parameter *k* represents the number of neighbors in the KNN (K-nearest neighbors) algorithm, $n_{x}$ and $n_{y}$, respectively represent the sample data falling within the range of the KNN, and N is the number of samples.

### 2.3. STN Prediction Models Based on Conventional Machine Learning Approaches

#### 2.3.1. Ordinary Least Square Estimation (OLSE) Regression

The linear regression is one of the most commonly used models, which is simple and easy to be built. Given a dataset of Vis-NIRS (marked as *x* = (*x_1_; x_2_; …; x_d_*)) with *d* wavelengths, the linear regression model of STN content can be presented as Equation (7).

(7) $$f\left(x\right)=w^{T}x+b$$

where w = (w_1_; w_2_; …; w_d_). The model is defined when *w* and *b* are obtained, and the most common approach to obtain *w* and *b* is to minimize the mean-squared error. The linear regression model was selected with the ordinary least square estimation (OLSE) in this paper.

#### 2.3.2. RF Regression

Random forest is an ensemble algorithm based on learning a collection of decision trees [32], and it is characterized by its ease of modeling, fast to calculate, and low computational overhead. RF builds several randomized decision trees at first, each of which is trained to classify the STN content. Then by using the decision tree as base learner, RF randomly chooses a specific number of wavelengths at each node and finds the best split among these wavelengths. Finally, RF generates predictions by voting over a collection of randomized decision trees.

#### 2.3.3. ELM Regression

Extreme learning machine is a kind of feed-forward neural network. Once the parameters (such as the weight between input layer and hidden layer, the threshold of the hidden layer) are determined, they do not change to adapt to new data. As a result, ELM performs fast-learning. In this paper, three-layer ELM was built, the number of hidden layer nodes ranged from 2 to 1000, and sine is selected as activation function in ELM.

### 2.4. STN Prediction Models Based on Convolutional Neural Networks

**Figure 2:**
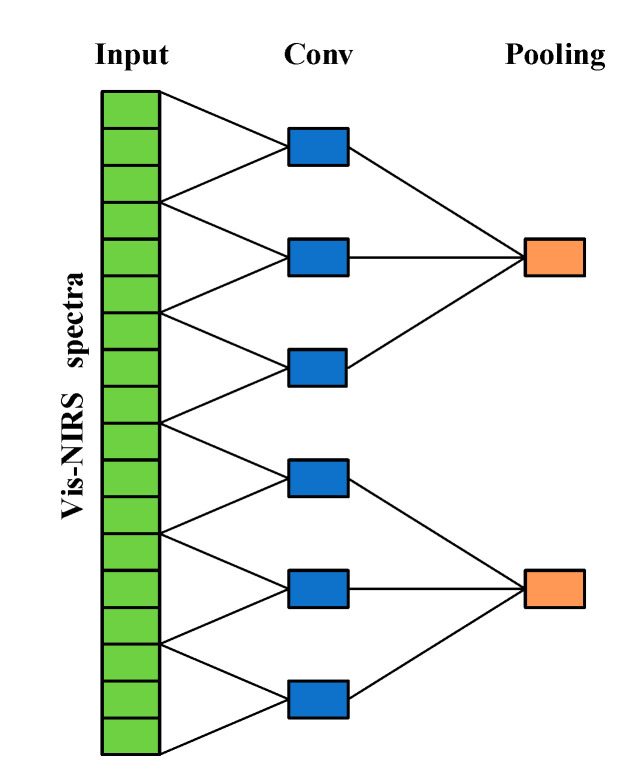
Feature processing of the receptive spectra region by one convolutional layer and one max pooling layer. This is an example of two layers with the convolutional layer having a filter size of three and stride of three and the max pooling layer having a pooling size of three. A green rectangle in the input layer represents one of the input spectral variables, a rectangle in the Conv or Pooling layer represents a neuron. One neuron in the pooling layer covers a receptive field of 9 original spectral variables, respectively.

Different from conventional machine learning, a convolution layer is used for feature extraction in CNN, together with a pooling to compress the features. Figure 2 represents an example of the feature processing with convolution and max pooling, the convolutional layers labeled as Conv and max pooling layer labeled as Pooling.

**Figure 3:**
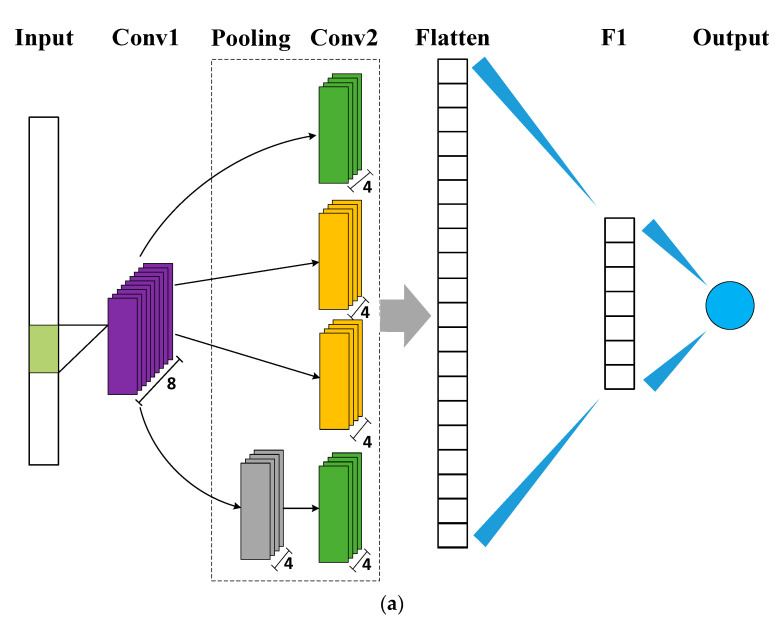

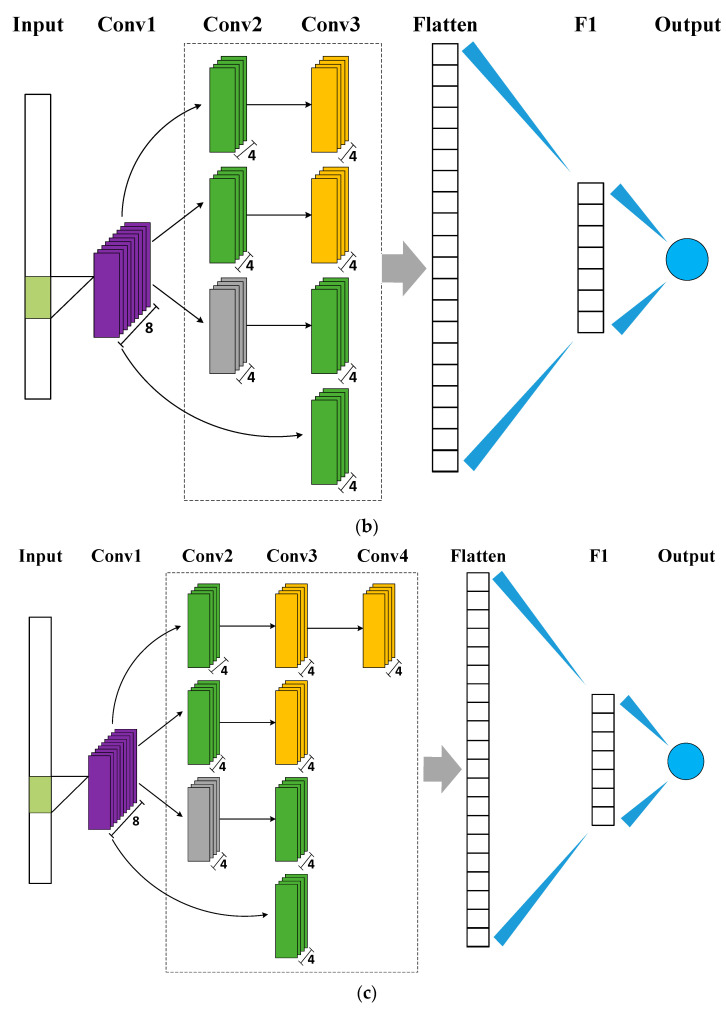
Architectures of three convolutional neural network (CNN) models incorporated Inception module. (**a**) Model 1. (**b**) Model 2. (**c**) Model 3.

The prediction effect of CNN can be affected by the structures of the networks. To evaluate the performance of different models, we built 3 STN predicted models incorporated Inception module [33], whose structures are shown in Figure 3. The major difference of these models lies in the numbers of 1 × 1 convolutions, which will impact the nonlinear fitting ability.

The input of each model is 4200-dimensional raw spectroscopic data and the output is the estimated STN content(s). The convolutional layers are labeled as Conv, flatten layer is labeled as Flatten, and fully connected layer is labeled as F1. The purple modules and yellow modules represent different sizes of general convolution to extract the feature. The green module represents 1 × 1 convolution to reduce the network parameters and enhance model fitting. The gray module represents max pooling (pool_size = 3) to compress the features. The first CNN model (Model 1) is the simplest one with introducing the minimum number of 1 × 1 convolution. The second CNN model (Model 2) and the third CNN model (Model 3) have the same number of 1 × 1 convolution, but model 3 is deeper than model 2. In this paper, zero-padding is used to retain the edge information, and the rectified linear unit (ReLU) is selected as the activation function for the convolutional layers and fully connected layer in each model. To search the local minimum of the objective function, we selected adam optimizer to train the model. Mean squared error (MSE) is adopted as the loss function, which is presented in Equation (8).

(8) $$\;Loss=\;\frac{\sum_{i=1}^{n}{\left(y_{i}-\hat{y_{i}}\right)}^{2}}{n}$$

where *y_i_* and $\hat{y_{i}}$ are measured values and predicted values, respectively. *n* is the number of samples in the training set, and *i* is the i-th sample. The detail hyperparameters set is shown in Table 2.

### 2.5. Model Evaluation

The model performance is evaluated by root mean squared error of prediction (RMSEP), the coefficient of determination (R^2^), and the residual prediction deviation (RPD), their calculations are shown in Equations (9)–(11).

(9) $$R^{2}\left(y,\hat{y}\right)=1-\frac{\sum_{i=1}^{n}{\left(y_{i}-\hat{y_{i}}\right)}^{2}}{\sum_{i=1}^{n}{\left(y_{i}-\bar{y}\right)}^{2}}$$

(10) $$RMSEP=\sqrt{\frac{\sum_{i=1}^{n}{\left(y_{i}-\hat{y_{i}}\right)}^{2}}{n}\;\;}$$

(11) $$RPD=\;\frac{s_{y}}{RMSEP}$$

(12) $$s_{y}=\sqrt{\frac{\sum_{i=1}^{N}{\left(y_{i}-\bar{y}\right)}^{2}}{N}\;}$$

where$\;y_{i}$ and $\hat{y_{i}}$ are measured values and predicted values, respectively; *n* is the number of samples in the training set; and $s_{y}$ is the standard deviation of the observed values, which is calculated using Equation (12). In which,$\;\bar{y}$ is the arithmetic mean of $y_{i}$; *N* is the number of samples in the test set. Note that in the Equation (9), R^2^ can vary from −∞ to 1 (i.e., it can yield negative values).

## 3. Results and Discussion

### 3.1. Impact of Preprocessing on Conventional Machine Learning Approaches

To study the effect of different pre-treatments on conventional machine learning approaches, different pre-treatments (including baseline correction (marked as Baseline), smoothing, dimensionality reduction (marked as Reduction), and feature selection (marked as Selection)) are adopted to pretreat the Vis-NIRS data. After preprocessing, the predicted models (including ordinary least square estimation (OLSE), random forest (RF), and extreme learning machine (ELM)) are built based on the new independent variable data, and the results are showed in Table 3.

By comparing the impact of different pretreatments to three modeling methods, we can conclude that baseline correction is optimal but MI feature selection performs worst. MI feature selection, as a preprocessing stage, is the method used to select a subset of relevant features for model construction. However, no subset can contain the full information of the raw spectral data. Thus, models built based on MI feature selection has the worst results among all models. PCA transforms the raw data into a lower dimensional orthogonal feature set while retaining maximum amount of information from the original high dimensional dataset. It preforms better than MI feature selection, but worse than the raw data without any pre-treatments. Baseline correction and smoothing filter the noise without reducing the information of raw data. Minor differences were observed between them with OLSE model, but baseline correction works better than smoothing with RF and ELM models. Both models perform better than that based on the raw data.

**Figure 4:**
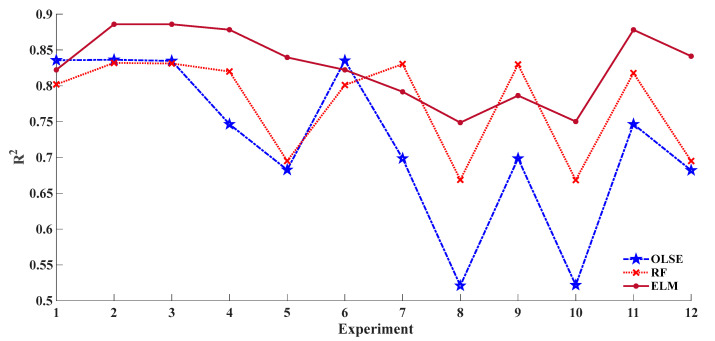
Stability of different predicted models with different preprocessing approaches.

Different prediction models with the same pre-treatment result in different performance. To observe and evaluate the stability of different models, the coefficient of determination (R^2^) for three models are plotted in Figure 4. As we can see in Figure 4, ELM showed the best adaptation and OLSE is the worst, this is due to non-linearity of ELM.

**Figure 5:**
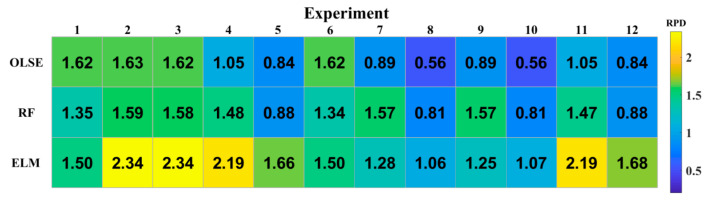
Usability of different predicted models with different preprocessing approaches.

To observe and evaluate the usability of different models, the RDP heatmap was plotted in Figure 5. If RPD is below 1.5, the model performance cannot be used for STN prediction due to poor performance. If it is between 1.5 and 1.8, the model needs to be improved to get better results. For values of RPD in the range of 1.8–2, the STN prediction model is considered to be practical, and if it is higher than 2.0, the model performance is considered to be very good [28].

For OLSE model, the RPD value is 1.62 with the raw data, but it decreases to 0.56 due to the MI feature selection. In this case, OLSE cannot be used for STN prediction. Baseline correction and PCA pretreatments can improve the availability of RF model, but the RPDs will be significantly reduced if MI feature selection involved. The best usability is obtained from the ELM. The RPD value is 2.34 with baseline correction, and can be used to prediction.

### 3.2. The Comparison among Three CNN Models and Conventional Models

To evaluate the model performance of different CNNs, the training process was repeated 25 times for each model. The results are shown in Table 4. Overall, CNNs based on raw data can obtain better results than conventional machine learning models. Model 3 provides the best prediction for STN (R^2^ is 0.93; RMSEP is 0.95 g/kg; and RPD is 3.85 in optimal case). The feature processing with convolution and pooling can retain the information of raw data, and it is not excised the features (or wavelengths). Meanwhile, nonlinear model (ELM) preforms better than linear model (OLSE), and the accuracy is improved by introducing nonlinear activation functions. So, among all the built models, the deep learning method shows encouraging support and the CNN model with deeper convolution layers illustrates the best performance among three different CNN structures built in this paper.

### 3.3. Impact of Data Properties on Prediction Models

Data properties, such as sample size, may have an impact on the predicted models. To study and reveal the impact, this paper reformed datasets with different data properties (Table 5) to analyze. What needs illustration is that these datasets are randomly sampled from raw dataset, in order to avoid the introduction of instrumental and experimental artifacts due to different collections.

**Figure 6:**
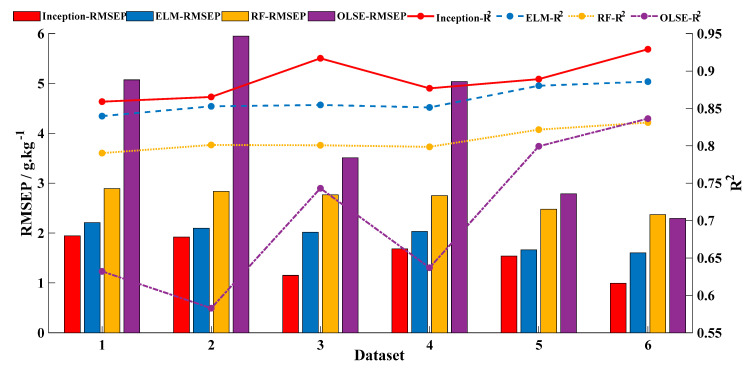
Performance of different predicted models with different size datasets. The models included baseline-corrected OLSE, baseline-corrected RF, baseline-corrected and smoothed ELM, and Model 3 of CNN. (For interpretation of the references to color in this figure legend, the reader is referred to the Web version of this article.).

According to the above modeling results, four models with different pre-treatments are selected to compare the performance on different data properties: baseline-corrected OLSE, baseline-corrected RF, baseline-corrected and smoothed ELM; and Model 3 of CNN. The performance of each model is shown in Figure 6. R^2^ is marked by the lines and RMSEP is marked by the histograms. Of which, Inception model is labeled as red, ELM model is labeled as blue, RF model is labeled yellow, and OLSE model is labeled purple.

**Figure 7:**
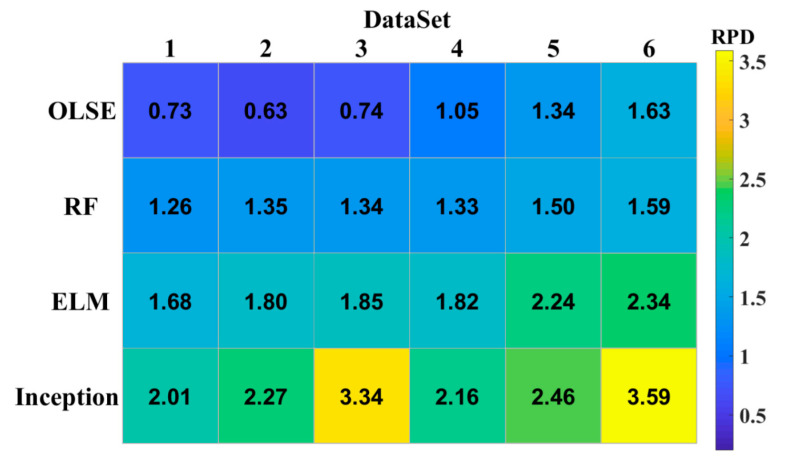
Effects of different predicted models with different size datasets.

The prediction accuracy of the models is poor with small sample data, and the accuracy improves as the amount of data increases. The effects of ELM and RF changed little on different datasets, but OLSE was fluctuated significantly. To evaluate the usability of different models based on different datasets, the RDP heatmap was plotted in Figure 7. Figure 7 shows that, as the amount of data increases, the value of RPD increases. The CNN incorporated Inception module performs better than the other models with different datasets.

**Figure 8:**
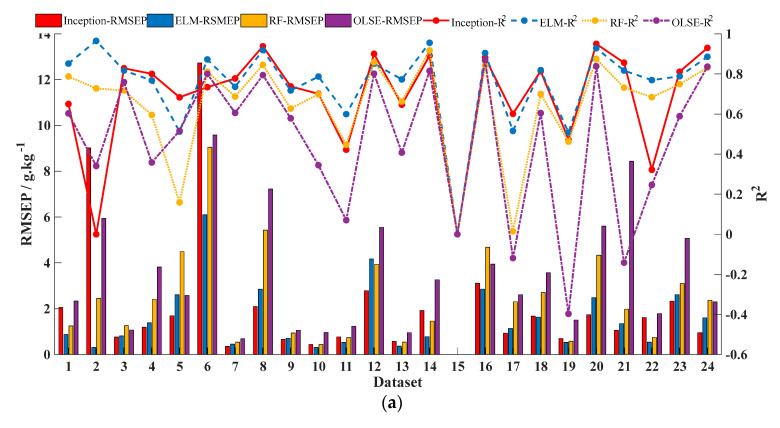

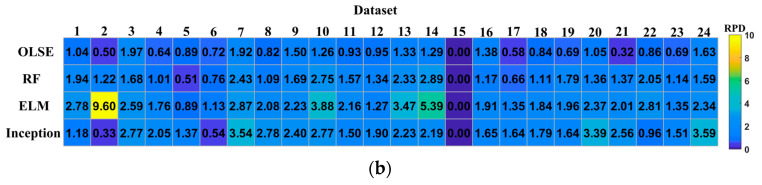
Effects of different predicted models with different datasets from different countries. (**a**) Accuracy of different predicted models. (**b**) Credibility of different predicted models.

In order to clarify the effectiveness and applicability of STN prediction models according to data characteristics, we separated the samples from different countries and made the new dataset (Table 6). Of these, France has the largest number of samples (2807 samples), and Luxembourg has the least number of samples (three samples). Based on the datasets of different countries, we compared the performance of different models (baseline-corrected OLSE, baseline-corrected RF, baseline-corrected and smoothed ELM, and Model 3 of CNN), the results are shown in Figure 8. The exceptional is that the results of the 15th dataset are set 0 due to the small number of samples.

The performance of the OLSE model is the worst among four models. RF model is predicted by dichotomy and presents some nonlinearity, but its performance needs to be improved. For the CNN incorporated Inception module, the accuracy depends on sample size and data distribution, and its credibility decreases as the sample size decreases. ELM model preforms the best in the four predicted models with the dataset divided based on different countries.

**Figure 9:**
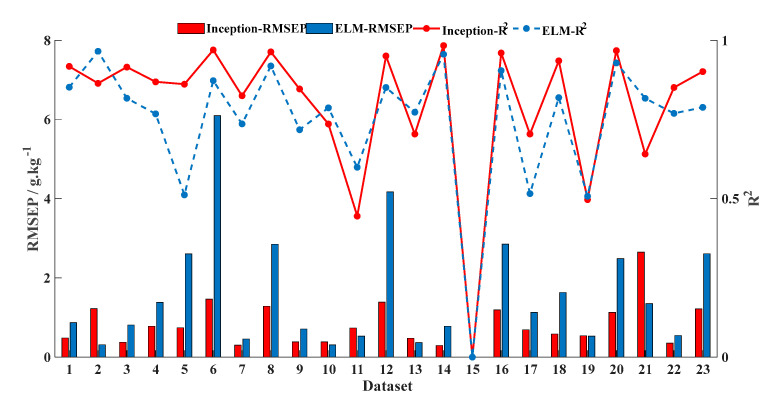
Effects of predicted with CNN and ELM.

From a practical point of view, the CNN model is always trained by the large size of samples and it can be used in each country soil samples. So, we selected the CNN model with total samples in optimal case (R^2^ is 0.93; RMSEP is 0.95 g/kg; and RPD is 3.85) to predict the STN content with different datasets from different countries, and compared with ELM model (Figure 9). Overall, the CNN model preforms better than ELM, but it is apparently worse in smaller size.

According to the research [34], a close relationship exists between organic carbon (OC) and N levels in soil. The higher OC concentration, the greater the N concentration. Moreover, the C-to-N ratio is relatively stable across different soil types. In order to investigate reliability of different STN prediction model, we divided the LUCAS dataset into 24 soil groups according to different OC concentrations by K-means clustering algorithm (Table 7), and the modeling results are shown in Table 8.

According to Table 8, the accuracy of each prediction model becomes worse based on OC content. We do not think it is because no obvious relationship exists between OC and STN; instead, it is because different countries have different agricultural production and farming histories, so that their soil physical structures and properties differ from each other. So, it is unreasonable and unscientific to divide data by OC concentration alone, it has to be combined country with OC concentration in order to get a high STN prediction accuracy model. Another conclusion can be drawn is that, due to the poor performance of OLSE, it should be noted that the original soil spectra does not have a linear relationship with the STN content.

### 3.4. Discussion

Agricultural production [35,36,37], soil structure [38], soil properties [39], soil sample data characteristics [40], along with the methods of data preprocessing [16,41], all affect the STN concentration prediction modeling and model performance. Due to the results we achieved in this paper, the STN prediction models with good performance and generalization come from the data set with greater size and more evenly distributed within a country. Data size [22] and data distribution [42] have a major impact on the predictive performance. The prediction accuracy of the model is poor with small sample data size and/or unevenly data distributed, and the accuracy improves as the amount of data increase and/or evenly distributed. Since different countries have different farming histories, and the soil physical structure differ with each other too, so that more suitable models can be built for each country, even for each different farmland in the country. According to the clustering results in this paper, we can see that C-to-N ratio is relatively stable across different countries since the distribution of STN content is following with OC concentration. However, because the data segmentation breaks the national boundaries, the predicted models perform significantly worse. It indicates that different types of soil from different country, even if the carbon content is in the same range, a robust STN prediction is hard to be built. Therefore, the ideal dataset is that include enough same type of soil samples with similar physical structure, evenly distributed and accurate STN concentrations and accurate soil spectra data. When this kind of ideal dataset is not available, then deep learning modeling approach would be very helpful.

As for the modeling method of soil total nitrogen concentration, both conventional and deep learning approaches can achieve practical accuracy due to this study. Data preprocessing method is very important to conventional modeling approaches [43]. In terms of data preprocessing, baseline correction is optimal and MI feature selection performs worst according to the results of different pre-treatments on conventional machine learning approaches. The reason for this is because MI feature selection has removed the useful information, and results in worse model performance. For deep learning approach, network construction is critical to the model performance. In terms of CNN structure, the predictive accuracy of CNN increases with the increase of the number of convolution layers due to the nonlinear activation functions. By modeling approaches comparing results, the deep learning method, CNN, provides good performance and robust generalization for STN content modeling, and it can be used as a benchmark model for all soil type and all countries as long as there are enough training samples from different soil type and different countries.

## 4. Conclusions

STN content prediction based on Vis-NIRS is becoming more and more feasible, but the performance of prediction is affected by various factors, such as soil properties, modeling method, characteristics of datasets, etc. In order to find a practical way to build STN content prediction model, three conventional machine learning methods and one deep learning approach are investigated and their predictive performances are compared and analyzed. Based on the results, the following conclusions are achieved:

(1) Misuse of preprocessing (such as feature selection) may introduce artifacts or remove useful patterns and result in worse model performance, but some preprocessing (such as baseline correction) may improve the results.
(2) Different data characteristics show different impact on modeling approach, so the ideal dataset is that include enough same type of soil samples with similar physical structure, evenly distributed and accurate STN concentrations and accurate soil spectra.
(3) The deep learning method, CNN, provides good performance and robust generalization for STN content modeling, and it can be used as a benchmark model for all soil type and all countries when there are enough training samples from different soil type and different countries. Otherwise, ELM is the best choice.

## Figures and Tables

**Table 1:** Description of the Land Use/Cover Area Frame Statistical Survey (LUCAS) Soil dataset.

Total Number of Samples	The Number of Countries	Method/Standard	STN (g/kg)		
			Min	Max	Mean
19,036	23	ISO 11261. 1995	0	38.6	2.92

**Table 2:** Hyperparameters set in three CNNs.

Hyperparameter	Model 1	Model 2	Model 3
Kernel size 1	9	9	9
Kernel size 2	5	5	5
Kernel size 3	7	7	5
Kernel size 4	---	---	7
Stride 1	3	3	3
Stride 2	2	2	2
Hidden number	32	32	32
Batch size	256	256	256
Dropout rate	0.4	0.4	0.4
Regularization coefficient	0.001	0.001	0.001
Learning rate	0.01	0.01	0.01
Learning rate decay	0.001	0.001	0.001

**Table 3:** The performance of three conventional models based on different pretreatment methods.

Model	Experiment No.	Baseline	Smoothing	Reduction	Selection	R^2^	RMSEP (g/kg)
OLSE	1	No	No	No	No	0.84	2.31
	2	Yes	No	No	No	0.84	2.30
	3	Yes	Yes	No	No	0.83	2.32
	4	Yes	No	Yes	No	0.75	3.56
	5	Yes	No	No	Yes	0.68	4.45
	6	No	Yes	No	No	0.84	2.31
	7	No	Yes	Yes	No	0.70	4.23
	8	No	Yes	No	Yes	0.52	6.69
	9	No	No	Yes	No	0.70	4.23
	10	No	No	No	Yes	0.52	6.71
	11	Yes	Yes	Yes	No	0.75	3.56
	12	Yes	Yes	No	Yes	0.68	4.46
RF	1	No	No	No	No	0.80	2.77
	2	Yes	No	No	No	0.83	2.37
	3	Yes	Yes	No	No	0.83	2.37
	4	Yes	No	Yes	No	0.82	2.51
	5	Yes	No	No	Yes	0.70	4.20
	6	No	Yes	No	No	0.80	2.78
	7	No	Yes	Yes	No	0.83	2.40
	8	No	Yes	No	Yes	0.67	4.64
	9	No	No	Yes	No	0.83	2.38
	10	No	No	No	Yes	0.67	4.63
	11	Yes	Yes	Yes	No	0.82	2.52
	12	Yes	Yes	No	Yes	0.70	4.20
ELM	1	No	No	No	No	0.82	2.49
	2	Yes	No	No	No	0.89	1.60
	3	Yes	Yes	No	No	0.89	1.60
	4	Yes	No	Yes	No	0.88	1.71
	5	Yes	No	No	Yes	0.84	2.25
	6	No	Yes	No	No	0.82	2.49
	7	No	Yes	Yes	No	0.79	3.00
	8	No	Yes	No	Yes	0.75	3.53
	9	No	No	Yes	No	0.79	3.00
	10	No	No	No	Yes	0.75	3.51
	11	Yes	Yes	Yes	No	0.88	1.71
	12	Yes	Yes	No	Yes	0.84	2.23

Note: Total samples of the dataset is 19,019, which the train dataset is 15,215 and the test dataset is 3804. ‘Yes’ indicates that this pre-treatment is used, and ‘No’ indicates this pre-treatment is not used.

**Table 4:** Predictive results obtained by three CNNs based on raw data.

Evaluation Metric	Model 1		Model 2		Model 3	
	R^2^	RMSEP	R^2^	RMSEP	R^2^	RMSEP
Mean	0.90	1.40	0.89	1.53	0.93	0.99
Std	0.013	0.182	0.014	0.193	0.003	0.037
Max	0.92	1.74	0.91	1.93	0.93	1.07
Min	0.88	1.15	0.86	1.33	0.92	0.95

**Table 5:** Description of the datasets with different size.

Dataset No.	Total Samples	Training Samples (Validation Samples)	Test Samples	Max	Min	Std
1	1903	1522 (381)	381	30.2	0.2	3.73
2	3801	3040 (760)	761	34.4	0.2	3.71
3	7609	6087 (1522)	1522	34.2	0.2	3.74
4	9508	7606 (1902)	1902	38.6	0.2	3.70
5	13,314	10,651 (2663)	2663	38.6	0.2	3.74
6	19,019	15,215 (3804)	3804	38.6	0.2	3.76

**Table 6:** Description of the datasets with different countries.

Dataset No.	Country	Samples	Max	Min	Average	Std
1	AUSTRIA	421	17.2	0.5	3.11	2.21
2	BELGIUM	71	12.4	0.9	2.89	2.54
3	CZECH REPUBLIC	407	24.2	0.5	2.16	1.9
4	GERMANY	1819	36.2	0.2	2.48	2.6
5	DENMARK	222	25.7	0.6	2.26	2.7
6	ESTONIA	219	34.4	0.6	5.67	7.21
7	SPAIN	2601	12.3	0.2	1.57	1.29
8	FINLAND	1662	32.3	0.2	5.08	5.9
9	FRANCE	2807	23.1	0.2	2.35	1.55
10	GREECE	487	9.1	0.2	1.59	1.14
11	HUNGARY	434	12.6	0.2	1.93	1.31
12	IRELAND	218	24.1	0.7	7.03	5.33
13	ITALY	1179	15.7	0.2	1.92	1.36
14	LITHUANIA	355	32.8	0.4	2.96	4.76
15	LUXEMBOURG	3	3.1	2.1	2.67	0.42
16	LATVIA	347	28.3	0.2	3.81	5.33
17	NETHERLANDS	200	18.6	0.3	2.3	2.22
18	POLAND	1610	38.6	0.2	1.9	3.17
19	PORTUGAL	475	7.6	0.2	1.67	1.1
20	SWEDEN	2232	34.2	0.2	5.04	5.91
21	SLOVENIA	112	19.7	0.8	4	2.77
22	SLOVAKIA	267	11.1	0.4	2.31	1.39
23	UNITED KINGDOM	871	27.3	0.4	4.03	3.3
24	TOTAL	19,019	38.6	0.2	2.93	3.76

**Table 7:** Description of the datasets with different organic carbon (OC) content.

Dataset No.	Samples	OC (g/kg)				STN (g/kg)			
		Max	Min	Average	Std	Max	Min	Average	Std
1	760	22.8	20.9	21.81	0.55	3.5	0.6	1.87	0.45
2	782	78.5	58.3	67.1	5.72	8.1	1.2	4.26	1.52
3	793	11.4	10.3	10.89	0.31	2	0.4	1.12	0.23
4	845	16.4	15	15.69	0.4	3.4	0.4	1.47	0.33
5	836	35.6	31.3	33.32	1.28	4.1	0.7	2.54	0.7
6	754	6.2	0	4.49	1.47	2.4	0.2	0.62	0.24
7	728	586.8	333.7	449.02	57.3	38.6	6	17.53	5.5
8	880	13.7	12.5	13.12	0.33	4.1	0.3	1.31	0.27
9	825	27.9	25.2	26.49	0.79	5.4	0.7	2.15	0.56
10	757	19.3	17.8	18.52	0.44	2.8	0.6	1.66	0.37
11	794	48.2	41	44.4	2.08	5.3	0.9	3.11	0.99
12	773	127.8	78.5	98.67	14.32	12.6	1.8	5.49	1.97
13	740	9.3	8	8.66	0.39	1.8	0.3	0.95	0.21
14	842	31.3	27.9	29.54	0.95	3.7	0.8	2.33	0.62
15	884	15	13.7	14.36	0.38	2.1	0.4	1.37	0.28
16	745	20.8	19.3	20.07	0.44	2.7	0.4	1.75	0.4
17	739	333.7	128.2	212.29	62.01	25.8	2.3	9.61	3.9
18	847	12.5	11.4	11.96	0.33	2	0.3	1.2	0.23
19	797	17.8	16.4	17.07	0.42	3.1	0.5	1.56	0.34
20	755	8	6.2	7.18	0.49	2.5	0.2	0.82	0.21
21	786	58.2	48.2	52.74	2.83	7.6	0.3	3.57	1.25
22	814	40.9	35.6	38.08	1.5	4.7	0.2	2.76	0.82
23	793	25.2	22.8	23.98	0.69	3.3	0.6	1.97	0.5
24	750	10.3	9.3	9.83	0.32	1.8	0.3	1.04	0.2

**Table 8:** Performance of different models with different datasets based on OC content.

Dataset No.	Inception			ELM			RF			OLSE			
	R^2^	RMSEP	RPD	R^2^	RMSEP	RPD	R^2^	RMSEP	RPD	R^2^	RMSEP	RPD	
1	0.69	0.06	7.17	0.63	0.07	6.07	0.48	0.10	4.31	−0.07	0.22	2.08	
2	0.63	0.87	1.76	0.71	0.69	2.23	0.44	1.31	1.17	0.35	1.52	1.01	
3	0.28	0.04	6.09	0.53	0.02	9.42	0.44	0.03	7.86	−0.38	0.07	3.20	
4	0.63	0.04	8.55	0.58	0.04	7.39	0.49	0.05	6.11	−0.47	0.15	2.12	
5	0.32	0.34	2.08	0.65	0.18	4.01	0.60	0.20	3.54	0.33	0.34	2.11	
6	0.09	0.05	4.80	0.28	0.04	6.05	0.15	0.04	5.15	−3.25	0.22	1.02	
7	0.79	6.26	0.88	0.79	6.27	0.88	0.52	14.51	0.38	0.59	12.30	0.45	
8	0.36	0.04	6.24	0.37	0.04	6.31	0.37	0.04	6.33	−1.40	0.15	1.65	
9	0.57	0.13	4.18	0.55	0.14	3.97	0.59	0.13	4.33	−14.99	4.99	0.11	
10	0.58	0.06	6.32	0.54	0.06	5.81	0.38	0.09	4.31	−1.12	0.30	1.26	
11	0.78	0.22	4.51	0.75	0.24	4.09	0.60	0.39	2.55	0.28	0.70	1.41	
12	0.73	1.08	1.84	0.65	1.38	1.43	0.39	2.38	0.83	0.06	3.69	0.54	
13	0.45	0.02	8.62	0.44	0.02	8.58	0.44	0.02	8.59	−1.28	0.10	2.10	
14	0.82	0.07	8.98	0.70	0.12	5.30	0.52	0.19	3.34	0.14	0.34	1.86	
15	0.44	0.05	6.24	0.49	0.04	6.85	0.47	0.04	6.58	−0.49	0.12	2.35	
16	0.09	0.15	2.75	0.63	0.06	6.68	0.52	0.08	5.18	−0.06	0.17	2.35	
17	0.67	4.96	0.78	0.49	7.65	0.50	0.31	10.23	0.38	−0.36	20.33	0.19	
18	0.29	0.04	5.95	0.56	0.02	9.52	0.50	0.03	8.40	−0.56	0.09	2.71	
19	0.28	0.08	4.08	0.59	0.05	7.15	0.56	0.05	6.64	−0.19	0.14	2.46	
20	0.29	0.03	7.23	0.37	0.02	8.19	0.05	0.04	5.42	−8.22	0.35	0.56	
21	0.82	0.30	4.29	0.81	0.32	4.02	0.58	0.70	1.84	0.54	0.76	1.68	
22	0.86	0.10	8.74	0.72	0.19	4.35	0.57	0.30	2.78	0.43	0.40	2.10	
23	0.64	0.09	5.40	0.58	0.11	4.68	0.49	0.13	3.83	−7.92	2.30	0.22	
24	0.19	0.03	6.06	0.54	0.02	10.59	0.51	0.02	9.93	−0.99	0.08	2.46	
